# Validation of a national checklist for continuous service improvement in a decentralised framework

**DOI:** 10.1093/eurpub/ckac129.430

**Published:** 2022-10-25

**Authors:** F Cardinali, S Carzaniga, L Martini, M Loiudice, F Carinci

**Affiliations:** 1 Quality, Safety and Good Practices, Italian National Agency for Regional Health Services, Rome, Italy; 2 Continuous Medical Education, Italian National Agency for Regional Health Services, Rome, Italy; 3 Department of Statistical Sciences, University of Bologna, Bologna, Italy

## Abstract

**Background:**

Previous studies allowed defining a novel checklist for the participatory evaluation of person-centredness in hospital care using 243 items, grouped in 4 main areas, 12 sub-areas and 29 criteria. We aimed to validate a reduced set of core items that could be continuously used for service improvement in Italy.

**Methods:**

Validation was performed using data collected during the last national survey carried out in 2017-2018 in N = 387 acute care hospitals from 16 out of 21 Italian regions. Descriptive measures for each item were used to assess eligibility for factor analysis, applied separately on each of the 4 main areas originally identified. Varimax rotation with eigenvalues>1 was used to optimise factor structure. Items with an item-total correlation>0.30 and factor loadings>0.4 were attributed to individual factors. Items with inter-item correlation coefficient>0.70 were included in a list of candidate mergers, submitted to expert opinion. Cronbach's alpha was used to assess overall internal consistency.

**Results:**

A total of 183 out of 243 items included in the original checklist were submitted to factor analysis. Overall values of Cronbach's alpha ranged between 0.77-0.90, indicating a high consistency. A total of 67 items were finally attributed to 4 main areas, allocated as follows: 16 items in 4 sub-areas for ‘Person-oriented organisational and care processes’, 16 items in 4 sub-areas for ‘Physical accessibility, livability and comfort of the facilities’, 15 items in 3 sub-areas for ‘Access to information, streamlining and transparency’, and 20 items in 4 sub-areas for ‘Taking care of the relationship with patients and citizens’.

**Conclusions:**

A simplified checklist including a manageable number of items that can be easily managed to evaluate hospital services was identified through an objective validation process. The national experience can provide valuable lessons for the application of participatory approaches of person-centred care.

**Key messages:**

• A standardised checklist has been validated using survey data collected through a participatory process in Italian regions.

• The checklist can be used to evaluate and improve person-centered hospital services through a manageable number of items and factors.

